# Alcoholic Stimulation

**Published:** 1896-08-01

**Authors:** Benjamin W. Richardson


### Medical Progress and Hospital Clinics.

*[The Editor will be glad to receive offers of co-operation and contributions from members of the profession. All letters should be addressed to* The Editor, at the Office, 428, Strand, London, W.C.]

### ALCOHOLIC STIMULATION.

By Sir Benjamin W. Richardson, M.D., F.R.S.

It has been my duty to dwell in these columns on the subject of alcohol as a remedy in disease, and I have tried to show that it can be dispensed with as a remedy in the most satisfactory manner, so that we can do very well without it. I have never, however, striven to show that alcohol is without its effects, and the subject before the profession is not simply alcohol as a remedy, but stimulation as a mode of cure; a subject that more than ever is being practically discussed.

In the days of the noted Dr. John Browne there was quite a fierce controversy upon alcohol. The Edinburgh School, with no less an authority than Cullen at its head, was averse to stimulation, and amongst the stimulants employed alcohol largely lost its hold. Browne made his appearance, and finding stimulation one of his methods of treatment, fell back upon alcohol, and got himself into trouble socially as well as medically. The Brunonian system was one of stimulation, and was so strong for the moment that it had its disciples and advocates. It set its author at variance with his brethren; he, in its defence, was obliged to administer his great remedy on—what may be considered as—"the sly," and got into scrapes from which he was, with difficulty, extricated. He made his theory, but he did not make a sound reputation with it. He certainly declared that alcohol was an antiseptic, and to that extent did good service, but he did not prove its practical value on the list of remedies. A patient suffered from fever, and Browne surreptitiously gave him, or had given to him, abundance of alcohol. Under the treatment the patient sank and died; a post-mortem was carried out, and the tissues of the body, dead as they were, seemed fresh to the eye and inodorous. Browne noticed their life-like character, and called attention to the circumstance that his treatment preserved the parts as if they were alive, a statement well deserving of notice and comment, as worthy of observation as are the evidences of a museum where the specimens are retained in spirit in a state of preservation.

The treatment of living matter and living function under alcohol is entirely different from the treatment of dead matter by it, and here lies the point that, as doctors, we have to decide upon and base our instructions. We can safely use alcohol as a preservative of dead substance, but can we use it or anything of the same class as safely as a stimulant on living material? In one instance we are recognising its true qualities; in the other we are using it for the sake of its vital action, and we cannot logically do both. Preservation, in so far as it means preservation merely, is freedom from decomposition, not from death. Life is active continued decomposition, but with reconstruction. If I touch an animal body with a thing that arrests action I touch its life and it must succumb; must become dead-drunk or dead-dead.

The more the profession, ignoring prejudice and old practices, think of stimulation as a mode of cure and argue it out, the better for the world. What is known as practice has proved nothing whatever in the matter. We have seen people dead-drunk recover consciousness, and we have seen sick people recover from their illnesses under alcohol, so that we have learned to look at the occurrence as natural, or even as more than natural, according to the law of necessity. But when we think over it we have seen persons, by the score, who have been well and hearty, though they have never been dead-drunk; and we have seen persons who have recovered from their sickness, although they never imbibed a drop of stimulant, and the fact that they get well under such circumstances is sufficient evidence that the stimulant has not been required. Men who are given to prescribe alcohol actually talk as if all persons under their care got well from the treatment, which, indeed, would be the case if they were right, whereas, under the alcoholic "sheet-anchor," the sick die in spite of it, and sometimes in sheer consequence of it.

Alcohol, in the end, does not stimulate, it really depresses if it does anything at all. We must admit it does something, and he is a mere fanatic who questions its effects. There can be no doubt that for a short time it whips on the circulation. It quickens the heart-beat; it increases the circulation of the blood; it excites the brain; it preserves the tissues from decomposition; but is it thereby of service? What it does seems to the profession, and feels to the sick, to be stimulation, and upon this all its assumed virtues rest. It appears to give new life; it raises the weak for a short time, even if it quickens death. It acts like running or drinking, or becoming mentally excited, and although no one would use these acts as remedies for disease, they might appear to have their advantages.

I do not agree with those who are astounded, and express their astonishment, when they see doctors giving wine or recommending spirits, wine, or beer to persons under their care. Life is a very slender thread; the doctor's art is very limited; the doctor is called to do; the people follow the doing, and constantly they feel stimulation render them service temporarily. If the doctor told them the truth, namely, that wine is the most detestable of mockers, they would not believe him, and so he is daily and hourly tempted to evoke phenomena which, from the beginning to the end, are a deceit; which shorten instead of prolong existence, and which may kill instead of cure. It is true, nevertheless, that every doctor should think for himself concerning the phenomena, and should prefer to rest on the permanent physiological, rather than on the empirical modes that lie at his disposal.

